# Supplementary material for: Are carbon emissions trading and green financial instruments synergistic? -Comprehensive quantitative research based on content analysis
Source: PLoS One. 2024 Mar 7;19(3):e0298601. doi: 10.1371/journal.pone.0298601 (PMC10919601; doi:10.1371/journal.pone.0298601)
Supplement: S1 Data — (ZIP) [file pone.0298601.s001.zip › Data/地级市Z_F工作报告.zip!/╣π╢1⁄2/╖≡╔╜╩╨/└┤╫╘╣1⁄2╓┌║┼í╛┬φ┐╦╩2╛▌═°í┐_2017.docx]

各位代表：

　　现在，我代表市人民政府，向大会报告政府工作，请予审议，并请各位政协委员和其他列席人员提出意见。

过去五年和2016年工作回顾

　　本届政府任期的五年，是佛山适应和引领经济发展新常态、转型发展开启新局面、城市综合竞争力不断提升的五年。面对复杂的经济形势和艰巨的改革发展任务，市政府在省委、省政府和市委的坚强领导下，在市人大、市政协的监督支持下，奋发有为、攻坚克难，扎实推进稳增长、调结构、促改革、惠民生、防风险各项工作，顺利完成各项目标任务，经济社会发展实现新跨越。

　　五年来，我们大力推进产业转型升级，经济综合实力不断迈上新台阶。地区生产总值从2011年6580.28亿元预计提高到2016年8600亿元，五年跨越两个千亿元台阶，年均增长8.5%；规模以上工业总产值从1.44万亿元提高到2.06万亿元，年均增长9.9%；全社会固定资产投资从1933.96亿元提高到3490.85亿元，年均增长12.5%；　社会消费品零售总额从1810.27亿元提高到3024.43亿元，年均增长10.8%；地方一般公共预算收入从341.73亿元提高到602亿元，年均增长12%。经济结构持续优化，三次产业比重由2011年1.9：62.1：36调整为2016年1.8：59.2：39。抢抓珠江西岸先进装备制造产业带建设机遇，加快打造万亿规模先进装备制造业产业基地，引进一汽大众、北汽福田、中国中车、千山药机等一批重大项目。制造业提质升级，成为国家制造业转型升级综合改革试点，依托智能制造推动产业结构优化升级获国务院通报表扬。民营工业对全市工业增长贡献率从2011年65.5%提高到2016年78.7%，美的、碧桂园跻身福布斯世界企业500强。创建国家创新型城市成效突出，全社会研发经费支出占地区生产总值比重从2011年1.9%提高到2016年2.62%，成为国家知识产权示范城市，佛山国家高新区获批创建珠三角国家自主创新示范区。

　　五年来，我们深入实施城市升级战略，城市功能品质不断提升。基本完成城市升级三年行动计划和两年延伸计划，投入2400多亿元建设444个项目，建成岭南天地、千灯湖公园、东平河滨江景观带等一批亮点工程，城乡面貌焕然一新。“一老三新”[1]强中心战略有效实施，“1+2+5+X”组团式现代化大城市格局初步形成。百村升级和历史文化街区保护成绩显著，全市村（居）基本实现“四整治一美化”[2]。“三旧”改造完成499个项目，用地面积2.61万亩，连续4次获得全省“三旧”改造考核一等奖。现代化综合交通体系逐步完善，“两环四纵五横”[3]高速公路网基本建成，轨道交通建设扎实推进，新增公路通车里程189公里，总里程达5387公里，中心城区公交分担率从2011年21%提升至2016年40.1%。水利、通信、能源等基础设施建设成效显著。城市精细化管理水平稳步提高，城市整洁度、美观度不断提升。成功创建全国文明城市。创建5A级旅游景区2个、4A 级旅游景区9个。生态环境持续优化，2016年二氧化硫年均浓度比2011年下降58.8%，PM2.5年均浓度比2013年下降28.3%[4]，首批整治的42条重点河涌达标率69%，新增公园绿地693.97公顷，市域森林覆盖率达35.5%，成为全国绿化模范城市。

　　五年来，我们持续深化改革开放，发展动力活力不断增强。深化行政体制改革，在全省率先编制实施市区两级政府部门权责清单，“一门式一网式”政府服务模式、商事登记制度、企业投资“三单”管理体制等改革取得阶段性成果。社会信用体系和市场监管体系建设全面展开。农村综合改革不断深化，“政经分离”“政社分开”加快推进，“三大平台”[5]促进农村资产管理逐步规范。获得设区的市地方立法权，建设完善区、镇（街）、村（居）三级公共法律服务实体平台767家，“一体两翼”[6]基层治理新格局初步构建。打造不是自贸区的“自贸区”，在全省率先上线试运行中国（广东）国际贸易“单一窗口”，“互联网+易通关”全面实施，成为全省法治化国际化营商环境试点城市。广佛同城化成效显著，两市交通基础设施加快对接，合作示范区建设扎实推进，产业、环保、民生等协作不断深化。积极主动推进广佛肇清云韶经济圈建设，加强粤港澳高端服务业合作，发起推动粤桂黔高铁经济带建设，区域一体化水平全面提升。企业“引进来”和“走出去”步伐加快，发起成立中德工业城市联盟，对外合作交流日益密切。

　　五年来，我们着力发展社会事业，民生福祉不断改善。预计全市财政民生支出累计达1829亿元，占一般公共预算支出的63.7%。建设人民满意政府“1+11”行动计划[7]全面实施，每年确定的省、市民生实事圆满完成，市图书馆、青少年宫、残疾人职业康复服务中心等一批民生项目启用。预计2016年城镇和农村常住居民人均可支配收入分别达4.3万元和2.4万元，年均增长10%和10.8%。就业形势保持稳定，新增就业42.8万人。社会保障水平稳步提升，社保覆盖范围不断扩大，2016年企业退休人员基本养老金月人均2875元，比2011年增长48.2%；城乡居保基础养老金月人均170元，比2011年增长70%。新建保障性住房2.8万套。城乡低保标准每人每月630元，比2011年增长68.5%；　农村五保供养标准月人均1535元，比2011年增长155.8%。教育累计投入815亿元，年均增长13.9%；　成为全省首个推进教育现代化先进市，义务教育加快向均衡优质化发展，新市民随迁子女就读公办学校比例保持70％以上。医药卫生体制改革不断深化，2016年每千常住人口医疗卫生机构床位数4.75张，比2011年增长34.2%；每千常住人口执业（助理）医师2.16人，比2011年增长17.4%，国家级卫生镇达19个，基本公共卫生服务水平和综合医疗卫生服务能力居全省前列。现代公共文化服务体系初步形成，万人拥有公共文化设施面积达2055平方米。公共体育设施建设加快，基本实现村（居）户外体育设施全覆盖。平安佛山建设成效明显，2016年刑事警情比2011年下降54.1%。安全生产监管“三大体系”不断完善[8]，2016年生产安全事故起数、事故死亡人数比2011年分别下降20.1%、5.1%。未发生较大以上食品药品安全事故。妥善应对自然灾害、疫情等公共突发事件。圆满完成对口帮扶云浮、清远“双到”任务。

　　五年来，我们全面加强政府自身建设，治理能力不断提高。自觉接受人大法律监督和政协民主监督，办理人大代表建议538件、政协提案991件，办复率100%。坚持依法行政，建立市长法律顾问制度，政府决策法治化规范化水平不断提升，政务公开深入推进，行政复议机制逐步完善，在省政府2015年度依法行政考评中名列首位。认真落实中央“八项规定”精神和国务院“约法三章”要求，扎实开展党的群众路线教育实践活动、“三严三实”专题教育和“两学一做”学习教育，从严从实整治“四风”问题，作风建设和廉政建设进一步加强。老年人、妇女儿童事业，审计、粮食储备、民族宗教、双拥优抚、台湾事务、档案方志、社会科学、人防、气象等工作不断取得新成绩。

　　2016年是本届政府任期的最后一年。我们坚决贯彻落实党中央、国务院，省委、省政府和市委的决策部署，积极应对各种压力挑战，全力推动经济社会平稳健康发展，实现“十三五”良好开局。

　　一是经济结构调整取得新成效。出台稳增长工作方案，预计2016年地区生产总值同比增长8.3%左右。实施扩大固定资产投资引领科学稳健发展三年行动计划，安排亿元以上项目713个，投资总额超1.5万亿元。完成工业投资1407亿元，比房地产开发投资高110亿元；　民间投资增长16%，占固定资产投资比重达72.9%。推动大型骨干企业跨越发展，新增年主营业务收入超100亿元企业4家，总数达16家；7家企业入围“2016中国民营企业500强”[9]。实施“百企智能制造提升工程”，带动近千家企业实施机器人及智能装备应用。开展“制造强国·佛山探路”活动　[10]，成功举办2016 Science机器人国际联盟大会　[11]。扶持发展“中国制造2025”试点示范企业，与珠江西岸五市共建“中国制造2025”试点示范城市群[12]。装备制造业完成增加值1470亿元，增长12%，其中“工作母机”增加值增长18%。圆满举办第二届珠江西岸先进装备制造业投资贸易洽谈会，引进科力远CHS、中铁华隧、美盈森[13]等一批优质项目。推动“两化”[14]深度融合，新增“两化”融合贯标试点企业46家，总数达103家，成功举办第二届中国（广东）国际“互联网+”博览会。召开特色小镇建设工作推进会，启动建设佛山国家高新区科技创新小镇群，北滘“智造小镇”入选全国首批特色小镇，张槎街道获批省级“互联网+”培育小镇。现代服务业发展提速，占第三产业增加值比重达58.5%。生产性服务业加快发展，佛山跨境电子商务公共服务平台上线试运行，广东金融高新区、广东省（佛山）软件产业园、广东工业设计城成为省级服务外包示范园区。旅游文化创意产业发展加快，罗浮宫国际家具博览中心成为首批国家工业旅游创新单位，南海影视城获评国家4A级旅游景区。

　　二是创新驱动发展实现新突破。把建设面向全球的国家制造业创新中心作为创新驱动发展的奋斗目标，出台创新驱动发展三年行动计划。狠抓高新技术企业培育，新增高新技术企业671家，总数达1388家，增长93.6%。科技创新平台数量稳步增长，建成新型研发机构30家，省级重点实验室17个、工程中心395家、技术中心150家；　规模以上工业企业研发机构、规模以上高新技术企业工程中心建有率分别达20%、85%。实施科技企业孵化器倍增计划，新增国家级科技企业孵化器6家，总数达10家；　新增国家级众创空间试点单位5家，总数达15家。完成工业技术改造投资550亿元，增长42.4%，总量稳居全省首位。深化产学研合作，启动近80项企业和产业关键核心技术攻关项目；与清华大学签订战略合作协议。佛山科学技术学院引进中国科学院院士、千人计划专家等高层次人才48人，录用博士109人，新校区建设进展顺利。实施重点产业人才引进培育暂行办法，全市新增市级以上创新团队19个，拥有国家“千人计划”专家41人。建设国家商标战略实施示范城市，拥有中国驰名商标157件，位居全国地级市首位。省市共建引领型知识产权强市启动。科技型中小企业信贷风险补偿基金累计帮助企业获得贷款授信20.46亿元。新增新三板挂牌公司38家，总数达79家；　新增私募股权投资基金55家，总数达334家。佛山海晟金融租赁股份有限公司获批运营，成为佛山首家金融租赁公司。广东金融高新区入驻金融机构和项目310家，总投资597.5亿元；股权交易中心注册登记企业2340家，帮助企业融资939.39亿元。

　　三是城市建设治理步入新阶段。召开城市治理大会，着力转变城市治理方式，加快城市治理体系和治理能力现代化建设。城市升级扩面提质，加快向城市升值转变。禅城区老城区活化成效显现，佛山古镇历史人文风貌逐步展现，绿岛湖片区水乡新城格局基本成型，南庄镇紫南村荣获“2016年中国十佳小康村”称号。南海区、狮山镇成为国家新型城镇化综合试点，里水镇艺术河畔、丹灶镇“一岛两湖”[15]建设成效明显，西樵镇松塘古村荣获“2016中国最美村镇传承奖”。顺德区北部片区一体化进程加快，广州大学城卫星城建设启动，市科学馆新馆、广东（潭洲）国际会展中心首期建成使用，东部片区总投资124亿元综合开发项目启动。高明区获评“最美中国·生态旅游目的地”，西江新城核心区一期基本建成。三水区北江新区建设加快，三水新城雏形初显。实施百村升级行动计划，完成30个古村落活化、30个城中村（旧社区）改造和48个“五好”新农村建设。广中江高速一期（荷塘至龙溪段）、魁奇路东延线二期通车，18条“断头路”打通[16]，“一环”西拓工程北环段和佛山地铁3号线开工。新建公共场所AP 接入点1.5万个，总数达3万个，全市光纤入户率达80%。新建地下综合管廊9.56公里，总数达24.85公里。基本完成100项环保民生实事，淘汰黄标车及老旧车2.15万辆。启动第二批90条“一河一策”重点河涌综合整治。完成100个村级工业区[17]环境整治，关停企业1221家。落实中央环保督察整改，分别立案处罚、关停取缔污染企业254家、161家。新建森林公园、湿地公园8个[18]，累计建成“绿城飞花”主题绿化景观49个，乡村绿化美化示范村达395个。

　　四是体制机制改革取得新进展。在全省率先出台供给侧结构性改革“1+5”工作方案[19]，出清国有“僵尸企业”105家，化解房地产库存476.33万平方米，为企业降低各类成本超280亿元；美的成为全国供给侧结构性改革先进典型；搭建众陶联，促进传统产业与互联网、金融资本融合发展，为陶瓷行业企业提供供应链集成服务；　创建全国质量强市示范城市，知名品牌示范区数量位居全国地级市首位[20]；弘扬工匠精神，命名30位“佛山·大城工匠”。深化“一门式一网式”政府服务模式改革，推进“两厅融合”[21]，全市“市民之窗”自助服务终端达1200台；清理规范68项行政审批中介服务事项，机动车跨区转移、抵押和注销登记等43项交管、车管业务实现同城通办。建立城乡社区建设工作联席会议制度，禅城区社会综合治理云平台入选2016年“互联网+政务”全国优秀实践案例。支持和培育各类社会组织发展，全市社会组织达6015个，数量位居全省地级市之首。深化国资国企改革，推动市属国有企业改革重组。南海区农村集体经营性建设用地入市改革试点工作有序开展。

　　五是区域开放合作迈出新步伐。与广州共同编制《广佛同城化“十三五”规划》，两市交通、产业等专项规划有效衔接。广州地铁7号线一期西延顺德段动工，广佛线二期通车。粤桂黔高铁经济带合作试验区纳入国务院《关于深化泛珠三角区域合作的指导意见》，上升为国家战略。落实粤港合作框架协议，成功举办2016香港·佛山节。对德合作进展顺利，成功举办2016中德对话论坛、中德企业投资与并购论坛、“中国制造2025”对话德国“工业4.0”大会等重大活动。帮助企业开拓国际市场，佛山泛家居品牌产品（伊朗）展示体验馆启动运营。鼓励企业参与“一带一路”建设，美的、东方精工等本土企业开展跨国并购，碧桂园开发马来西亚森林城市项目，联塑打造全球销售服务平台“领尚环球之家”。落实6.03亿元财政扶持资金，新时期精准扶贫对口帮扶湛江、云浮工作开局良好，援疆援藏、对口四川凉山州扶贫协作、支援联系四川甘孜州工作扎实推进。

　　六是社会民生事业取得新成绩。人民满意政府建设行动计划完成150个年度重点项目，根据第三方机构评估结果，2016年佛山市建设人民满意政府得分85.18分，比2015年提高1.37分，市民对政府公共服务满意度排名全省第一。全市人社系统发放创业担保贷款2.4亿元，带动3.4万人就业。启动基本医疗保险城乡一体化改革，统一全民医保待遇。城乡低保对象和低保临界对象大病医疗救助比例达90%以上。成为国家学前教育改革发展实验区、国家特殊教育改革实验区，公益普惠性幼儿园占总数75%以上，普通高中优质学位达100%，城乡同步实施免费中等职业教育。启动卫生强市建设，成为公立医院综合改革省级联系试点，58.4万居民签订家庭医生服务协议，基本公共卫生服务均等化考核排名全省第一。中心城区“两横四纵”公交骨干线路开通运营，新增公交专用道39.4公里，总里程达141.2公里。创建“城乡十分钟文化圈”示范镇（街）5个、示范村（居）15个，建设提升150个行政村（社区）综合文化服务中心。成功举办2016广东非遗周暨佛山秋色民俗文化活动，市工人文化宫改造项目竣工。入选全省首批足球试点城市。连续第八次荣获“全国双拥模范城”称号。成为创建国家食品安全示范城市试点，在全省地级市政府食品安全评议考核中名列首位，市级食品安全示范点达1400个，“明厨亮灶”[22]餐饮单位达4536家。严厉打击四类刑事犯罪[23]，在全省“飓风2016”专项行动综合考评中排名第一。安全生产标准化企业达2.35万家，达标总数和比例居全省首位，安全生产“五个重大突破”[24]经验在全省推广。制定全国首个新市民服务发展专项规划和全省首个《居住证实施办法》，在全省率先出台户籍制度改革方案，大幅拓宽新市民入户渠道，新增户籍人口11.21万人。

　　各位代表！过去五年，我们始终坚持在传承历届市委、市政府发展战略思路的基础上，结合新形势新要求不断改革创新，努力探索实践具有佛山特色的转型升级之路。我们深切体会到：做好佛山的工作，走好佛山的路，必须坚持以制造业为立市之本、强市之基，做优做强做大实体经济；必须坚持放心放胆放手发展民营经济，增强经济发展的内生性、根植性和稳定性；必须坚持创新驱动，推动发展方式从要素驱动向创新驱动转变，加快形成以创新为引领和支撑的经济体系和发展模式；必须坚持开放引领，全面扩大对内对外开放，加快形成空间更大、层次更高、内涵更深的开放合作新格局；必须坚持深化改革，着力破除制约发展的体制机制障碍，不断释放改革红利；必须坚持以高水平规划引领城市现代化，以城市现代化促进产业高端化，提升城市发展质量和综合竞争力；必须坚持以人为本，以人民满意为评价标准和发展导向，努力建设人民满意政府，不断增强人民群众的幸福感和获得感。

　　各位代表！我市过去五年改革发展成绩来之不易，这是在省委、省政府和市委正确领导下，在市人大、市政协监督支持下，全市人民团结拼搏、锐意进取的结果。在此，我代表市人民政府，向全市广大干部群众，中央、省驻佛山单位，驻佛山人民解放军指战员、武警官兵和社会各界人士表示崇高的敬意！向长期关心支持佛山发展建设的港澳台同胞、海外侨胞、国内外友好人士表示衷心的感谢！

　　我们也清醒地认识到，我市经济社会发展仍然存在不少困难和问题，主要是：有效需求增长乏力，有效投资强度偏低，新的经济增长点不多，经济增长面临较大下行压力；制造业总体效益和产业层次水平不高，自主创新能力不强，产业转型升级任重道远；资源环境约束加剧，土地开发强度较高，大气、水污染治理及生态建设压力较大、任务艰巨；城市管理科学化、精细化水平还有较大提升空间；　民生社会事业存在不少短板，优质公共服务供给不足；一些政府工作人员主动作为、责任落实不够，使命意识、担当意识、法治意识有待增强。对此，我们必须高度重视，切实加以解决。

今后五年奋斗目标和主要任务

　　未来五年，是佛山站在新的历史起点上，抢先谋划第二个百年目标，开启率先基本实现社会主义现代化新征程的重要战略机遇期。放眼全球，世界经济处于深度调整期，新一轮科技革命和产业变革孕育兴起，国际竞争和分工格局正发生深刻变化。综观国内，经济下行压力依然较大，但总体呈现缓中趋稳、稳中向好态势，新旧发展动能加快转换，经济结构调整持续深化，我国经济发展长期向好的基本面没有变。立足佛山，我们虽然面临不少挑战和考验，但我市坚守实体经济，制造业基础好、民营经济实力强、市场环境优，经济平稳健康发展的根基牢固；同时，国家制造业转型升级综合改革试点扎实推进，珠三角国家自主创新示范区、珠江西岸先进装备制造产业带、粤桂黔高铁经济带加快建设，广佛携手打造珠三角湾区世界级城市群核心[25]，为我市经济社会又好又快发展增添了机遇和动力。我们必须坚定发展信心和战略定力，增强责任意识、机遇意识、使命意识，主动适应和引领经济发展新常态，为全省经济增长和结构调整发挥支撑作用，奋力开创佛山改革发展新局面。

　　今后五年政府工作的总体要求：全面贯彻党的十八大、十八届三中、四中、五中、六中全会精神，坚决维护以习近平同志为核心的党中央权威，深入贯彻落实习近平总书记系列重要讲话精神，按照“五位一体”总体布局和“四个全面”战略布局，牢固树立创新、协调、绿色、开放、共享发展理念，按照党中央、国务院，省委、省政府和市委的决策部署，以新发展理念为引领，以提高发展质量和效益为中心，以全面深化改革为根本动力，以创新驱动发展为核心战略，以推进供给侧结构性改革为主线，加快产业转型升级，提升城市功能品质，构建高水平开放型经济新格局，建设绿色生态美丽家园，全面增进民生福祉，在率先基本实现社会主义现代化新征程中走在前列。

　　今后五年经济社会发展主要奋斗目标：地区生产总值年均增长7.5%以上，人均地区生产总值年均增长7%；　居民人均可支配收入年均增长7.5%以上；　全社会研发经费支出占地区生产总值比重达3%；完成“十三五”规划发展目标，全面建成高水平小康社会和国家创新型城市，建设面向全球的国家制造业创新中心、具有全国影响力的制造业转型升级示范城市、宜居宜业宜创新的高品质现代化国际化大城市、更具品质的文化导向型城市、更高质量的民生幸福城市。

　　为实现上述目标，必须处理好以下五个关系：

　　——处理好稳增长与调结构的关系。以稳增长为调结构创造空间，以调结构为稳增长夯实基础，在着力保持经济中高速增长的同时，更加注重培育新的经济结构，增强新的发展动力，促进经济平稳健康可持续发展，努力推动产业结构迈向中高端。

　　——处理好存量优化与增量优质的关系。既要倍加珍惜、充分发挥传统制造业存量优势，以科技创新、技术改造等途径促进其优化提升；　也要加快发展先进制造业、战略性新兴产业、现代服务业等优质增量，推动新产业、新业态尽快上规模、上档次。

　　——处理好需求侧与供给侧的关系。围绕破解经济结构性失衡问题，在充分发挥投资、消费、出口等需求侧对经济增长拉动作用的基础上，把经济工作的重心转移到供给侧上来，着力振兴实体经济，扩大有效供给，提升供给质量，强化供给结构对需求结构的适应性。

　　——处理好城市、产业、文化与人的关系。推进以人为核心的城镇化，全面优化城市功能，挖掘提升历史文化内涵，促进文化与城市、产业相融共生，努力实现生产空间集约高效、生活空间宜居适度、生态空间山清水秀，让市民在优美环境中安居乐业。

　　——处理好经济发展与社会建设的关系。牢固树立以人民为中心的发展理念，把满足人民群众物质文化需求、提高人民群众生活水平作为经济发展的最终目的，在坚持以经济建设为中心的同时，加快推进以改善民生为重点的社会建设，不断增加优质公共服务供给，让发展成果更多更公平惠及市民群众。

2017年工作安排

　　2017年是新一届政府开局之年，也是实施“十三五”规划的重要一年。我们将认真贯彻市第十二次党代会的决策部署，坚定信心、稳中求进，努力推动经济平稳健康发展与社会和谐进步。

　　今年经济社会发展主要预期目标：全市地区生产总值增长8.0%~8.5%，人均地区生产总值增长7%以上；地方一般公共预算收入增长9.5%；全社会固定资产投资增长15%；社会消费品零售总额增长10%；进出口总额力争增长1%；居民人均可支配收入增长与经济增长基本同步；城镇登记失业率控制在3.5%以内；居民消费价格涨幅控制在3%左右；单位地区生产总值能耗、主要污染物排放量下降幅度完成省下达任务。

　　为实现上述目标，重点做好以下八方面工作：

　　一、努力保持经济平稳健康发展，持续增强经济实力

　　坚持稳中求进工作总基调，内挖潜力，外拓市场，构建内外需协调拉动经济增长新格局，用心服务扶持企业发展，确保经济平稳较快增长。

　　着力扩大有效投资。落实扩大固定资产投资引领科学稳健发展三年行动计划，全年安排项目建设644个，计划投资2100亿元，持续抓好产业、交通、能源等重大项目建设，提高投资的有效性和精准性。加快推进省、市重点项目建设，完善项目督查服务机制，全年计划完成投资720亿元。实施新一轮技术改造，落实工业技改事后奖补政策，鼓励企业扩产增效和设备更新，力争全年实现工业技改投资增长20%。促进民间投资健康发展，放宽民间投资准入，引导社会资本通过PPP、BOT、委托运营[26]等方式，参与基础设施、生态环保、社会民生等项目建设。

　　积极培育消费需求。主动适应消费结构升级趋势，扩大优质商品和服务有效供给，加快培育养老、教育、文化、体育等新消费热点。挖掘住房、汽车、家电、餐饮等传统消费潜力，支持居民自住和改善型住房需求。加强商业街区和商贸中心规划建设，打造一批特色鲜明、错位发展、功能完备的区域性商圈。促进网络与信息消费，加快发展电子商务，培育一批综合性电商龙头企业和行业性电商平台。创建省放心消费试点城市，优化市民消费环境。

　　促进外贸回稳向好。稳步推进“互联网+易通关”改革，加快建设国际贸易“单一窗口”，提升贸易便利化水平。加快发展跨境电商等外贸新业态，扶持培育一批高水平外贸综合服务企业，力争成为国家泛家居市场采购贸易方式试点。发挥各级外贸扶持专项资金、中小微企业投保出口信用保险专项资金作用，探索推进国通保税物流中心（B型）升级综合保税区建设，支持企业积极应对国外反倾销反补贴等措施，推动外贸出口稳定增长。

　　支持民营经济做优做强做大做成“百年老店”。落实促进民营经济发展政策措施，常态化开展暖企行动，切实帮助企业解决实际困难。落实产权保护制度，依法维护民营企业合法财产和权益。积极培育大型骨干企业，引导民营企业通过技术升级、主业扩张、兼并重组、上市等方式实现跨越发展，力争全年新增主营业务收入超100亿元企业2家。鼓励中小企业向“专精尖”方向提升，培育更多细分行业“单打冠军”。支持行业协会（商会）发展，建设中小企业公共技术服务平台，增强服务企业能力。引导民营企业推广应用精益生产方式，走以质取胜、品牌强企之路。弘扬新时期佛山企业家精神，加强对企业家特别是新一代民营企业家的培养，推动民营企业稳健传承。

　　二、深化供给侧结构性改革，促进经济加快转型升级

　　坚持把供给侧结构性改革作为经济工作的主线，促进制造业提质增效和转型升级，不断提高供给体系质量和效率。

　　深化供给侧结构性改革。落实供给侧结构性改革“1+5”工作方案，深入推进“三去一降一补”。积极稳妥去产能。做好国有“僵尸企业”出清工作，着力清理整治“三高一低”[27]企业，有序推动产业梯度转移。因地制宜去库存。构建购租并举的住房保障制度，严格控制商业地产开发规模，引导商业办公项目用地功能调整，鼓励库存商业地产改造为创新创业孵化器、众创空间以及现代服务业载体，探索建立房地产平稳健康发展长效机制。坚守底线去杆杠。加快发展多层次资本市场，拓宽企业直接融资渠道，完善处置非法集资防控机制，加强金融风险防控。真金白银降成本。继续降低制度性交易成本，减轻企业税费、社保、物流等成本负担，鼓励传统行业发展供应链集成服务。注重实效补短板。加快推进补齐软硬基础设施短板14项重点工程[28]。积极创建全国质量强市示范城市，弘扬“匠心铸精品、质量强佛山”理念，全面提升佛山制造美誉度。实施品牌带动战略，打造一批有特色、有规模、有影响力的区域品牌。大力实施标准化战略，支持龙头骨干企业、行业协会、标准联盟参与和主导国内国际标准制定。深入推进农业供给侧结构性改革，增加绿色优质农产品供给，提高农产品标准化生产、品牌创建和质量安全监管水平。

　　推动制造业智能化高端化发展。以智能制造为主攻方向加快制造业转型升级，创建一批“中国制造2025”试点示范企业，力争先进制造业和高技术制造业增加值增长不低于10%。着力打造全国机器人自主创新集成中心，实施“机器引领”计划和机器人及智能装备生产应用“百千万”工程[29]。探索制定智能产品技术标准，推进智能泛家居产业标准化建设，提升家电、陶瓷卫浴等智能产品竞争力。发挥珠江西岸先进装备制造产业带龙头作用，抓好科力远CHS、一汽大众二期、北汽福田二期等重大项目建设。强化“大招商、招大商”理念，优化招商引资体制机制，瞄准先进装备制造、“工作母机”、战略性新兴产业、现代服务业等领域开展精准招商，着力引进一批投资超10亿元，技术含量高、经济效益好、带动能力强的重大项目。

　　提高现代服务业发展水平。大力发展工业设计、现代物流、商贸会展等生产性服务业，引导制造业企业延伸发展产品设计、技术开发、系统控制等增值服务，加快由生产制造型向生产服务型企业转变。以广东（潭洲）国际会展中心为依托，办好珠江西岸先进装备制造业投资贸易洽谈会、中国（广东）国际“互联网+”博览会等重大展会，争取中国国际中小企业博览会落户，打造佛山会展品牌。发挥广东工业设计城等平台作用，加快工业设计产业集聚发展，打造“工业设计之都”。推进国家物流标准化试点城市建设，提升流通网络化、智能化、信息化水平。加快发展旅游休闲、文体娱乐、教育培训、健康养老等生活性服务业，让都市生活更加便利、更为宜居、更有品质。积极申报省文化金融合作试验区，推动中国·佛山石湾陶瓷创意谷创建国家级文化产业示范园区，禅城区、高明区创建省全域旅游示范区，加快华侨城文化旅游综合体、美的·鹭湖森林度假区、三水尼克文化生态海岸及大旗头广府文化基地等项目建设，培育一批具有较强影响力和竞争力的文化企业，促进旅游文化创意产业加快发展。

　　培育发展“四新经济”。大力发展新产业、新业态、新模式、新技术，积极培育经济新增长点。全面实施“互联网+”行动计划，推动新一代信息技术与各行业深度融合，培育发展线上线下体验式消费、共享式消费和个性化定制服务，规范发展网络购物、约车及订餐等新业态，构建分布式、扁平化、分享型产业生态链。顺应“万物互联、跨界融合”发展潮流，总结推广众陶联、维尚家具、林氏木业等企业发展模式经验，引导制造企业通过协同设计、协同制造、协同服务，加快产品、业态、模式和服务创新，增强产业链供应链上下游协同响应能力。以禅城区建设广东大数据综合试验区为示范，培育发展云计算、大数据、物联网等产业，着力构建完整的大数据产业体系。

　　三、实施创新驱动发展战略，着力打造国家制造业创新中心

　　坚持把科技创新作为引领发展的第一动力，围绕打造国家制造业创新中心“三步走”[30]战略目标，不断聚集科学发展新动能。

　　加快推进珠三角国家自主创新示范区建设。实施创新驱动发展三年行动计划，全力创建国家创新型城市，着力打造以“一环”沿线佛山国家高新区、乐平智能创新示范园、佛山国家火炬创新创业园、顺德北部片区创新走廊等载体为节点的“一环创新圈”，构建区域自主创新发展新格局。推进佛山国家高新区跨越发展，力争2018年、2020年分别进入全国前30名、前20名，推动顺德高新区申报国家级高新区。加快国家知识产权示范城市建设，加强知识产权质押融资风险补偿资金管理，积极申报中国（佛山）先进装备制造业和建材知识产权保护中心，严厉打击知识产权侵权行为。

　　着力推动大众创业万众创新。加快“互联网+”创新创业示范市建设，高标准打造“丰收街·菁创聚”双创社区等平台，优化创新创业环境，激发创新创业活力。牢牢扭住高新技术企业这个“牛鼻子”，大力培育和引进高新技术企业，力争两年内高新技术企业达2000家，入库后备企业超过700家。实施科技企业孵化器倍增计划，完善孵化育成体系，力争2017年科技企业孵化器总数达50家，其中国家级孵化器13家。推动规模以上工业企业建立研发机构，支持企业建立研发准备金制度，力争规模以上高新技术企业、工业企业建有研发机构率分别达95%、25%。推动政产学研用协同创新，深化与中国科学院、中国工程院、清华大学、卡内基梅隆大学等国内外科研院校战略合作，组织开展核心技术和关键共性技术攻关。加快推进佛山科学技术学院建设高水平理工科大学，鼓励开展“高校＋高端研究院所＋龙头企业”产学研合作，着力将其打造成为全球创新技术应用中心、高新技术企业的大孵化器，以及培养应用型工程师和企业家的“摇篮”。

　　深化金融科技产业融合发展。围绕产业链部署创新链，围绕创新链完善资金链，通过政策链实现系统融合，构建科技金融资源高效对接的体制机制。综合运用科技发展基金、创业投资基金、风险补偿基金、财政贴息贷款等方式，鼓励企业加大科技创新投入，引导社会资本参与科技创新。大力发展创业投资、风险投资、股权投资和金融租赁，设立市创业投资引导基金，支持上市公司、龙头企业参与设立创投、并购基金和产业基金，打造珠江西岸创投中心和融资租赁区域中心。积极申报国家产融合作试点城市，加强广东金融高新区和“互联网+”众创金融示范区建设，争取成为国家级金融改革创新平台。加快发展总部金融，支持组建民营银行，完善金融组织体系。用好100亿元创新创业产业引导基金、15亿元企业融资专项资金等扶持资金，大力推广“政银保”、政策性小额贷款保证保险，帮助企业缓解融资困难。

　　构筑创新人才支撑体系。以舍得投入、舍得时间、舍得声誉的气魄抓好人才工作，健全人才政策，创新扶持方式，引进培育一批科技领军人才、高水平创新团队、行业技能人才和管理人才。加快佛山科学技术学院新校区建设，确保秋季顺利招生。支持佛山职业技术学院、顺德职业技术学院建设一流职业院校。深化与省属驻地高校合作，支持南海大学城及南方医科大学（顺德校区）、广东财经大学（三水校区）等发展，推进国家和省研究生联合培养基地建设。加快建设广州大学城卫星城。

　　四、提高城市治理水平，建设宜居宜业宜创新的高品质现代化国际化大城市

　　将以人为本理念融入城市规划建设管理全过程，实施城市治理“1+8”三年行动计划[31]，加快实现从城市升级向城市升值，再向城市治理现代化的大跨越。

　　强化城市规划引领。健全城乡规划编制体系，建立以城市总体规划为统领、近期建设规划为抓手、控制性详细规划为核心的城乡规划体系。加快推进“三规合一”[32]编制工作，打破部门条块分割，建设国土规划管理平台和数据中心，构筑全市规划国土管理综合“一张图”。启动城乡规划条例立法工作，进一步理顺市、区、镇（街）三级规划管理体制，实行城乡规划统一编制年度计划制度，实现“一张蓝图干到底”。严格依法执行规划，加强规划审批后跟踪监督。

　　从容推进城市建设更新。遵循城市发展规律，以建设质量和安全为前提，有序开展城市建设和城市更新，提高城市综合承载能力。完善“两环四纵五横”高速公路网络，推进广佛肇高速佛山段、佛江高速北延线、佛清从高速佛山段等项目建设。实施“断头路”连通五年行动计划，年内打通20条“断头路”。加快“一环”西拓工程北环段建设，启动南环段建设。推动轨道交通“四网合一”[33]，加快佛山地铁2号线一期、3号线、南海新交通试验线、高明现代有轨电车示范线等项目建设，做好2号线二期、4号线一期、9号线一期、11号线、13号线一期等项目前期工作并力争年内动工，确保佛山西站主站场投入使用，推进轨道站点周边用地TOD 综合开发。全力治理交通拥堵，深化TC公交[34]改革，推进中心城区“十纵十横”公交骨干线网[35]建设，推行一站式智能化辅助公交服务，提升中心城区公交分担率。加强城市充电桩和加氢站基础配套建设，年内各区建设1座加氢站、配套1条氢能源公交车示范运营线路。加强海绵城市建设，重点推进绿岛湖片区等7个区域试点[36]，新建项目全面落实海绵城市建设要求，加快地下综合管廊建设。实施城市更新三年行动计划，抓好省新一轮深化“三旧”改造综合试点。加强水利、通信、电力等基础设施建设，确保220千伏熙悦输变电等工程按期投产。

　　统筹城乡协调发展。以镇村现代化促进城市现代化，加快城市功能板块优化整合，完善提升“强中心、多组团、网络型”城镇空间格局，提高佛山新型城镇化水平。依托城市中轴线和东平河水轴线，打造城市发展脊梁和滨水生活长廊，推动“一老三新”融合发展，提升中心城区辐射带动能力和优质服务功能。建设提升狮山新城、顺德北部片区、西江新城、三水新城等片区，推动城市组团差异化联动发展。加快建设一批产业特色鲜明、生态环境优美、人文气息浓厚的美丽特色小（城）镇，力争全年创建15个市级特色小镇。着力推进“美丽文明村（居）”和省级新农村连片示范建设，实施古村落活化升级延伸计划，力争全年创建“五好”新村居112个，推动城市基础设施、公共服务和现代文明向村（居）辐射、延伸和覆盖。深入推进村级工业区综合整治，引导传统产业园区向现代产业社区转变。

　　实施精细化城市管理。统筹推进城市管理和服务，提高数字化管理水平，打造统一信息共享平台，创新城市精细化管理模式。推进智慧城市建设，加快实施智能交通、智能警务、智慧教育、智慧医疗等基础工程和民生服务智慧项目。建设光网城市和无线城市，推动光纤网络和新一代移动通信基站加快发展，新增公共场所AP接入点5000个，光纤入户率超83%。强化城市管理考评，加强对城市道路、市政设施、景观照明、园林绿化的维护管理，推进沿街景观提升，集中开展居民小区等重点区域违法建设专项整治。加快制定完善城市管理领域地方性法规和地方政府规章，确保城市管理有法可依。

　　五、加快推进基础性关键性改革，再创佛山改革发展新优势

　　勇于先行先试，进一步深化基础性关键性改革，努力实现有效市场和有为政府的有机统一，把改革红利转化为发展新动能。

　　深化经济领域改革。以国家制造业转型升级综合改革试点为契机，在财税体制改革、金融创新、土地制度改革、扶持企业发展等方面积极探索，着力构建有利于制造业发展的体制机制。积极主动对接广东自贸区，围绕投资便利化、贸易便利化、金融创新等重点领域，复制推广自贸区改革经验做法，营造与国际贸易规则体系相适应的制度环境。全面深化国有企业改革，完善国资监管体制，推动混合所有制改革和国有资产证券化，引导国有资本重点投向水电气、交通基础设施、金融、文化旅游等公共服务和战略性新兴产业领域，推动国有企业建立以董事会建设为核心的现代企业制度，激发国有经济活力和动力。

　　加大“放管服”改革力度。深化行政审批制度改革，推进行政审批标准化建设，着力精简创新创业、民生服务等重点领域行政许可事项。加快“互联网+政务服务”发展，完善全市统一的“一门式一网式”政府服务模式改革，提升网上、手机终端、自助终端办事深度和效率。健全企业投资“三单”管理制度，深化商事登记制度改革，推广跨部门并联审批联办机制，扩大法人“一门式”综合服务事项。加快建设政民互动大平台，提升12345平台综合服务水平。完善社会信用体系建设，推动市场监管体制创新。做好承担行政职能事业单位改革、公共资源交易平台整合等试点工作。

　　推进农村综合改革。深化农村产权制度改革，加快农村土地承包经营权确权登记颁证工作，逐步形成农村土地所有权、承包权、经营权分置格局。统筹协调推进农村土地征地制度改革与农村集体经营性建设用地入市改革试点，健全程序规范、补偿合理、多元安置的农村土地征收制度，探索建立城乡统一的建设用地市场。健全集体资产交易管理制度，完善农村集体资产交易、财务网上监控、股权管理等信息化平台，促进农村集体经济组织健康发展。深化村务、居务公开，健全村（居）议事协商民主机制，促进基层社会民主协商、共建共治。探索吸纳非户籍常住人口进入村（居）“两委”等基层组织，积极参与基层社会治理。做好村（居）“两委”换届选举工作。

　　深化社会体制改革。加快全国社区治理和服务创新实验区建设，优化社区服务设施布局，提升基层治理网格化服务管理水平。加强社会组织和社工人才队伍建设，引导社会组织在参与社会治理、提供社会服务、整合社会资源等方面发挥积极作用。抓好综治信访维稳，排查化解社会矛盾，健全落实“三官一师直联”[37]“一村居一专职调解”机制，推动基层治理法治化。加强社会事业重点领域改革创新，实施现代职业教育综合改革示范市、国家特殊教育改革实验区、国家学前教育改革发展实验区等教育综合改革；　深化医药卫生体制改革，开展公立医院综合改革、基层医疗卫生机构综合改革，促进社会办医加快发展。

　　六、加强区域交流合作，推动高水平双向开放

　　以“一带一路”战略引领佛山通达世界，将区位优势转化为区位责任，争当珠三角湾区世界级城市群建设主力军，着力构建对内对外开放合作新格局。

　　提高对外开放合作水平。扎实推进“一带一路”建设，深化与沿线国家的经贸、科技、人文交流合作，着力打造“一带一路”战略先行区。鼓励支持优秀企业通过并购重组、股权置换、境外上市、项目建设等方式“走出去”。实施佛山泛家居品牌产品海外展示体验馆建设三年行动计划，力争年内在澳大利亚等地设立场馆，促进佛山品牌产品走向国际市场。积极吸引利用外资，引导外资更多投向高新技术制造业、高附加值服务业。深化友好城市交流合作，发挥中德工业服务区、广东国际合作区[38]、中德工业城市联盟等平台作用，推进中欧城镇化合作示范区建设，不断提升城市国际化水平。

　　加快广佛同城化建设步伐。以广佛同心携手打造珠三角湾区世界级城市群核心为引领，重点推进南海—荔湾、顺德—番禺、三水—花都等合作示范区建设。加强两地智能装备、汽车制造等产业协作，共同参与全球要素配置、产业分工和市场竞争。推动构建广佛轨道交通“一张网”，加快广州地铁7号线一期西延顺德段、珠江大桥放射线接广佛新干线等跨界交通基础设施建设，打造连接两市核心区的快速干道，推动佛山与广州白云国际机场、广州南站、广州南沙港等全面对接。以广佛跨界区域水环境整治为重点，加强生态环境共治，深化两地文化教育、社保医疗、社会治理、政务服务等领域合作，推动民生服务对接共享。

　　推动区域合作发展。积极融入粤港澳大湾区[39]发展战略，主动承接深圳创新溢出效应，加大技术、资本、人才等对接合作力度；深化与港澳地区在科技创新、先进制造业、现代服务业等领域合作，加快粤港澳合作高端服务示范区建设，继续办好香港·佛山节。积极参与广佛肇清云韶经济圈建设，加大佛山、云浮产业共建力度，推进佛山（云浮）产业转移工业园等“两园六区”[40]建设。加快粤桂黔高铁经济带合作试验区（广东园）建设，加强与高铁沿线城市合作发展。做好对口支援西藏墨脱和新疆伽师、对口四川凉山州扶贫协作、支援联系四川甘孜州工作，扎实开展湛江、云浮精准扶贫。强化重大发展规划、重大基础设施建设、重点产业布局、重点领域和关键环节等市级统筹力度，推动产业资源、基础设施适度向高明区、三水区倾斜，促进全市区域协调一体化发展。发挥各区比较优势，发扬比学赶帮超的干事创业精神，构建各区开放竞争、协作并进、合作共赢发展新格局。

　　七、注重生态环境保护，促进绿色可持续发展

　　坚持绿色低碳循环发展，突出绿色富市、绿色惠民，加快建设天蓝、地绿、水清的美丽佛山。

　　强化环境综合治理。构建“党政同责、一岗双责”环境保护责任体系，打好大气、水、土壤污染防治三大攻坚战。加快推进第二批90条重点河涌及建成区黑臭水体整治，全面推行河长制，实施全流域综合整治，确保广佛跨界河流水质达到省考核要求。着力加强细颗粒物、二氧化氮、臭氧等污染物控制，推动重点区域和行业污染综合治理，确保完成100家VOCs　排放重点监管企业整治，提升扬尘污染和高排放车辆管控水平，加强汽车尾气治理，切实提高包括氢能源汽车在内的城市新能源汽车推广比例，稳步改善空气质量。实施农用地和建设用地土壤环境分级分类管理，推进土壤污染治理和修复试点示范工作，启动南海绿色工业服务中心建设。推动环保行政执法与刑事司法相衔接，对各类环境违法行为零容忍、零弹性、零余地。

　　加大生态保护力度。积极创建国家森林城市，推动珠三角国家绿色发展示范区建设。持续实施新一轮绿化佛山大行动，开展“绿城飞花”景观、生态景观林带、森林进城围城、乡村绿化美化等绿化工程，确保建成森林公园3个、湿地公园4个，新造林绿化面积1900公顷以上。加快潭洲水道生态景观长廊（陈村段）、东平河北岸景观亮化等工程建设，构建开放舒适、生态宜人的滨水空间体系。开展土地利用总体规划调整和全域性永久基本农田划定，落实高标准基本农田建设任务。大力发展现代都市型农业，打造7个现代农业重点园区[41]，建设1000亩以上生态健康养殖示范小区，着力构建万亩桑基鱼塘生态系统，重塑岭南水乡风貌。

　　节约高效利用资源。实行能源和水资源、建设用地总量和强度双控行动，推动能源管理体系、企业能源管理中心、能效对标体系建设。建立覆盖所有固定源的排污许可证管理制度，开展排污权有偿使用和交易。探索绿色小区、绿色城区、绿色城镇建设，大力推广装配式建筑，推动绿色建筑规模化、产业化、标准化。加快分布式光伏、环保装备、环保服务等产业发展。开展节约型园林绿地建设，实施城市绿地分级养护管理。加快生活垃圾减量化、资源化。

　　八、着力保障和改善民生，共建和谐幸福家园

　　把保障和改善民生作为改革发展的出发点和落脚点，坚持守住底线、突出重点、加大投入，努力让市民群众拥有更多幸福感和获得感。

　　提高基本公共服务水平。加强创业带动就业，加快全市“1+5”创业孵化基地建设，完善高校毕业生、异地务工人员、就业困难人员就业服务体系，城镇新增就业7.8万人。加强养老服务设施规划，构建功能完善、覆盖城乡的养老服务体系，不断提升养老服务水平。继续提高企业退休人员基本养老金和城乡居保基础养老金标准，推动老年优待服务向常住佛山的外地户籍老人覆盖。完善低保、特困人员供养等特殊困难对象救助制度。提升优抚保障水平，完善复退军人服务体系。加强卫生强市和健康佛山建设，完善分级诊疗制度，家庭医生签约服务覆盖率不低于30%。加大公立医院基础设施和重大医疗设备投入，力争两年内建成6类重大医学科技创新平台、15所高水平医院、60个高水平医学重点专科，社区卫生服务机构建设达标率达100%。实施“全面两孩”生育政策，加强计划生育服务管理。推进义务教育优质均衡一体化发展，实施义务教育阶段学校基础设施五年提升行动计划，五年内新增义务教育阶段学位18万个，其中公办学位15万个。加大教育经费保障力度，幼儿园生均公用经费财政补助标准年内提高至每生每年不低于1000元。完善新市民积分制，推动外来常住人口市民化，促进新市民更好地融入佛山。

　　加快建设文化导向型城市。实施“文化佛山”三年行动计划，着力打造“世界美食之都”“世界功夫之城”“陶艺之都”等系列文化品牌。落实岭南特色街区复兴计划，加强对梁园、仁寿寺、康有为故居等历史文化街区和历史建筑的保护活化。注重传统文化保护与利用，促进非物质文化遗产传承与发展，继续办好佛山秋色巡游、佛山功夫嘉年华等重大活动。创建国家公共文化服务体系示范区，加快佛山国际体育文化演艺馆、佛山新城文化中心等文化基础设施建设，深入开展“佛山韵律·和风鸣畅”等艺术惠民活动，推动“文化惠民”向“文化悦民”升级。加强公共体育设施建设，开展50公里徒步等群众体育活动，办好第九届市运会、佛山国际功夫搏击赛、佛山国际龙舟赛、世界狮王争霸赛、欧洲高尔夫球挑战巡回赛佛山公开赛等赛事。

　　深入推进平安佛山建设。建立食品安全“党政同责、一岗双责”责任机制，全力创建国家食品安全示范城市，完善食用农产品质量安全快速检测体系，提升食品药品基层监管能力，加大食品药品违法犯罪打击力度。深化“中心+网格化+信息化”社会治安防控体系建设，推动刑事、交通、火灾等警情持续下降。坚决落实安全生产责任制，完善城市风险点、危险源排查常态化治理体系，深化对交通运输、建筑施工、油气管道、消防等重点行业领域安全生产专项整治，抓好村级工业区安全生产整治提升工作。加强流动人口及出租屋服务管理。强化三防管理工作，提高突发事件应急处置能力。

　　认真办好十件民生实事。一是建设义务教育学校34个，新增义务教育阶段学位2.7万个以上。二是实施基本医疗保险城乡一体化改革[42]。三是提高城乡居民最低生活保障标准，惠及2.8万困难群众。四是开展“健康细胞”工程建设，建成40个健康村（居）、社区、医院、学校等。五是完善公交配套设施建设，新建公交专用道30公里，新增、更新公交车300辆和公共自行车1000辆。六是全面实施100项环保民生实事。七是建设300公里污水管网、16项民生水务重点工程。八是新增、改造公园绿地面积193公顷。九是建设行政村（社区）综合文化服务中心300个。十是按时保质保量完成省下达的住房保障工作任务。

　　各位代表！做好今年经济社会发展各项工作，必须加强政府自身建设，不断提升政府治理能力。我们要进一步增强“四个意识”[43]特别是核心意识、看齐意识，贯彻落实《中国共产党党组工作条例（试行）》等各项党内法规，坚持在市委领导下开展工作。一是坚持依法行政。以创建珠三角法治政府示范区为契机，强化法治政府建设，推进重点领域政府立法，完善政府立法程序和公众参与政府立法机制，确保2018年率先实现法治政府建设目标。加强政府法律顾问队伍建设，推动重大行政决策程序规范化。自觉接受人大及其常委会监督，积极支持政协履行政治协商、民主监督、参政议政职能，办理好人大代表建议和政协提案。推动执法体制机制改革创新，提高综合执法水平，加强行政复议规范化建设。深入开展“七五”普法工作，落实“谁执法谁普法”责任，在全社会形成浓厚法治氛围。二是主动有效作为。深入开展“两学一做”学习教育，严格落实“三个区分”，研究建立容错免责机制，鼓励各级领导干部“为官有为”，从严整治不作为、慢作为、乱作为及庸政懒政怠政。认真落实领导干部挂钩联系工作机制。完善政府绩效管理和评价机制，强化年度目标任务考核。三是加强作风建设。强化对厉行节约、公务接待、职务消费等规定执行情况的常态化监督，坚决防止“四风”问题反弹。深入推进廉洁试验区建设，坚持惩治腐败“无禁区、全覆盖、零容忍”。完善党风廉洁建设大数据平台，健全监察、审计、司法联动监督机制，推进审计机关人财物管理改革，强化行政权力监督。

　　各位代表！站在新的历史起点上，面对跨越发展新机遇，我们责任重大、使命光荣！让我们紧密团结在以习近平同志为核心的党中央周围，在省委、省政府和市委的正确领导下，真抓实干，创新进取，为实现市十二次党代会确定的宏伟目标，开创我市改革开放和现代化建设新局面而努力奋斗！
